# ﻿Molecular phylogeny and taxonomy of the *Hydrangeaserrata* complex (Hydrangeaceae) in western Japan, including a new subspecies of *H.acuminata* from Yakushima

**DOI:** 10.3897/phytokeys.188.64259

**Published:** 2022-01-12

**Authors:** Shun K. Hirota, Tetsukazu Yahara, Kengo Fuse, Hiroyuki Sato, Shuichiro Tagane, Shinji Fujii, Tadashi Minamitani, Yoshihisa Suyama

**Affiliations:** 1 Field Science Center, Graduate School of Agricultural Science, Tohoku University, 232–3 Yomogida, Naruko-onsen, Osaki, Miyagi 989–6711, Japan Tohoku University Osaki Japan; 2 Kyushu Open University, 744 Motooka, Fukuoka, 819–0395, Japan Kyushu Open University Fukuoka Japan; 3 The Kagoshima University Museum, Kagoshima University, 1-21-30 Korimoto, Kagoshima, 890–0065, Japan Kagoshima University Kagoshima Japan; 4 Department of Environmental Science, University of Human Environments, Okazaki, Aichi, 444–3505, Japan University of Human Environments Okazaki Japan; 5 Tsunehisa 5-4-7, Miyazaki 880–0913, Japan Tsunehisa 5-4-7 Miyazaki Japan

**Keywords:** cpDNA, DNA barcoding, *F _ST_*, island, ITS, MIG-seq, threatened plants

## Abstract

According to the contemporary classification of *Hydrangea* native to Japan, *H.serrata* is a polymorphic species including six varieties. We discovered a plant identified as *H.serrata*, but morphologically distinct from previously known varieties, in Yakushima island where approximately 50 endemic species are known. To determine the relationship of this plant with previously known varieties, we examined morphology and constructed a highly resolved phylogeny of *H.serrata* and its relatives using three chloroplast genomic regions, *rbc*L, *trn*L intron, *psb*A-*trn*H, and two nuclear genomic regions, ITS1 and ITS2, and Multiplex ISSR genotyping by sequencing (MIG-seq). Based on these morphological and phylogenetic observations, we describe Hydrangeaacuminatasubsp.yakushimensis**subsp. nov.** as a newly discovered lineage in Yakushima, Japan and propose *Hydrangeaminamitanii***stat. nov.** and Hydrangeaacuminatasubsp.australis**stat. nov.** which were previously treated as varieties of *H.serrata*.

## ﻿Introduction

*Hydrangea* L. s. lat. is a genus of Hydrangeaceae, comprising approximately 200 species distributed in East and Southeast Asia and the New World (De Smet et al. 2015). Based on molecular phylogenetic studies, De Smet et al. (2015) proposed a broad circumscription of *Hydrangea* by absorbing the other eight genera of tribe Hydrangeeae. Under this proposal, *Cardiandra* Siebold & Zucc., *Deinanthe* Maxim., *Pileostegia* Hook. f. & Thomson, *Platycrater* Siebold & Zucc., and *Schizophragma* Siebold & Zucc., which have been recognized in the representative flora of Japan (Kitamura and Murata 1979; Satake et al. 1999; Ohba 2017), are reduced to *Hydrangea* s. lat. In contrast, Ohba and Akiyama (2016) preferred to retain these genera and proposed generic segregation of most of the sections and subsections of *Hydrangea* s. lat. proposed by De Smet et al. (2015). In this study, we follow the broad circumscription of *Hydrangea* by De Smet et al. (2015) that retains species widely known as “hydrangea,” including *H.macrophylla* (Thunb.) Ser. and *H.serrata* (Thunb.) Ser., under the genus name of *Hydrangea*.

In 2005, we discovered a plant of the genus *Hydrangea* from a mountain-top area of the Yakushima Island, a small island with an area of 504.88 km^2^ and a maximum peak of 1,936 m in elevation, part of which is designated as a UNESCO Man and the Biosphere Reserve (Okano and Matsuda 2013). The Yakushima Island is a center of plant endemism in Japan, harboring approximately 45 endemic species, including *Hydrangeagrosseserrata* Engl. (Masamune 1934; Hotta 1974; Yahara et al. 1987). Whereas *H.grosseserrata* grows in evergreen forests at lower elevations, the newly discovered plant of *Hydrangea* is restricted to the mountain-top. In addition, they are morphologically distinct from *H.grosseserrata*. Although the flora of Yakushima has been well studied by the classic work of Masamune (1934) and a subsequent work of Yahara et al. (1987), recent field surveys discovered six additional new species endemic to this island: *Oxygyneyamashitae* Yahara & Tsukaya (Burmanniaceae, Yahara and Tsukaya 2008), *Carexmochomuensis* Katsuy. (Katsuyama 2009), *Haplopterisyakushimensis* C.W. Chen & Ebihara (Pteridaceae, Chen et al. 2014), *Dryopterisprotobissetiana* K. Hori & N. Murak. (Dryopteridaceae, Hori et al. 2015), *Lecanorchistabugawaensis* Suetsugu & Fukunaga (Orchidaceae, Suetsugu and Fukuhara 2016), and lastly *Sciaphilayakushimensis* Suetsugu, Tsukaya & H. Ohashi (Triuridaceae, Suetsugu et al. 2016). Considering the high endemism of the flora of Yakushima, we suspected that the plant of *Hydrangea* could be a new taxon. In this study, we compared the newly discovered plant with a morphologically similar species by molecular phylogenetic analysis and morphological observations.

The newly discovered plant is morphologically identified as *Hydrangeaserrata* in having ovate-oblong petals, distinct peduncles, and oblong leaves, based on the key and description of Ohba (2017). According to Yamazaki (2001) and Ohba and Akiyama (2013), *H.serrata* is a polymorphic species, including six varieties, but the plant discovered from a mountain-top area of the Yakushima Island appeared to be different from those varieties. Among these six varieties, the following three varieties are distributed on the main island of Kyushu located 60 km north of Yakushima: H.serratavar.acuminata (Siebold & Zucc.) Nakai, var.australis T. Yamaz., and var.minamitanii H. Ohba. To examine the genetic divergence of the newly discovered plant from the three varieties of *H.serrata* distributed on the Kyushu Island, we reconstructed phylogenetic trees of *H.serrata* and its relatives using three chloroplast genomic regions, *rbc*L, *trn*L intron, *psb*A-*trn*H, and two nuclear genomic regions, ITS1 and ITS2, and Multiplex ISSR genotyping by sequencing (MIG-seq; Suyama and Matsuki 2015).

A previous molecular phylogenetic study was performed on *H.serrata* and its relatives using *rbc*L, *mat*K, and Random Amplified Polymorphic DNA (RAPD) markers (Uemachi et al. 2014), but this study did not examine var.australis and var.minamitanii. Uemachi et al. (2014) revealed that H.serratavar.serrata diverged to the western and eastern groups in Japan, corresponding to H.serratavar.acuminata and H.serratavar.serrata s. str., respectively.

Our new molecular phylogenetic analysis covered all the lineages distributed in Kyushu, including the newly discovered lineage from Yakushima, H.serratavar.acuminata , var.australis , and var.minamitanii from western Japan, as well as var.angustata (Franch. & Sav.) H. Ohba and var.serrata s. str. from eastern Japan. The results supported the treatment of the former three varieties as H.acuminatasubsp.acuminata, H.acuminatasubsp.australis, and *H.minamitanii*, respectively, and treating the newly discovered lineage as a new subspecies of *H.acuminata*.

## ﻿Materials and methods

### ﻿Field surveys

**Figure 1.**
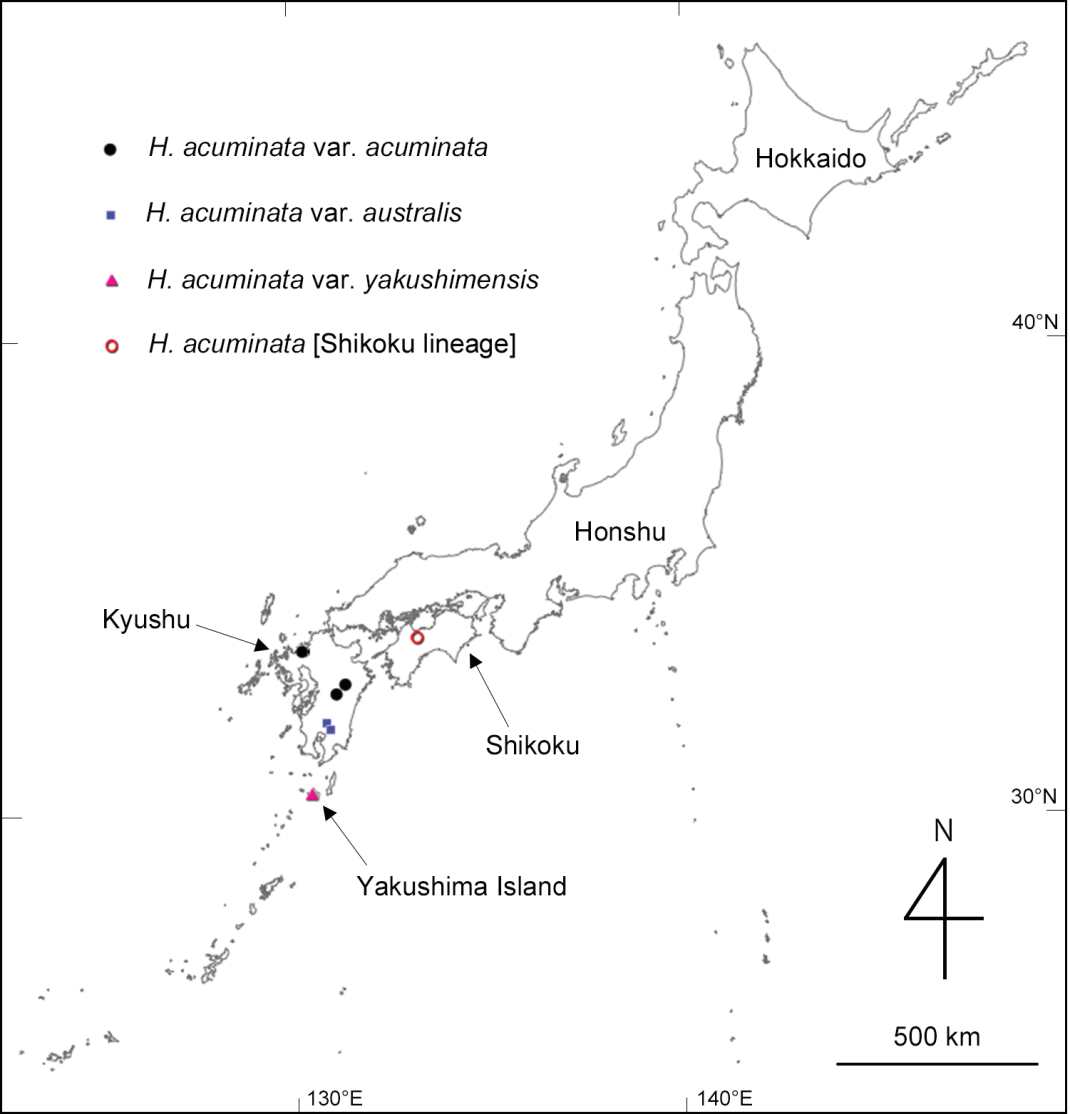
Localities of Hydrangeaacuminatasubsp.acuminata (including Shikoku lineage), subsp.australis , and subsp.yakushimensis where DNA samples and voucher specimens were collected in this study. The map was produced from Chiriin Chizu Vector (https://maps.gsi.go.jp/vector/).

We carried out field studies in Yakushima Island of Kagoshima Prefecture and five additional prefectures, including Fukuoka, Miyazaki, Kochi, Mie, and Shizuoka. In total, we collected 24 samples consisting of 10 species with five infraspecific taxa of *Hydrangea* for DNA isolation (Table 1): H.acuminatasubsp.acuminata from four localities (Fig. 1), H.acuminatasubsp.australis from two localities (Fig. 1), H.acuminatasubsp.yakushimensis described below (Fig. 1), *H.macrophylla*, *H.minamitanii*, H.serratavar.angustata, and H.serratavar.serrata of sect.Macrophyllae (E. M. McClint.) Y. De Smet & Samain (De Smet et al. 2015); *H.grosseserrata*, *H.kawagoeana*, *H.luteovenosa*, and *H.scandens* of sect.Chinenses Y. De Smet & Samain; and *H.hirta* of sect.Hirtae Y. De Smet & Samain. These three sections belong to the monophyletic group Hydrangea II (De Smet et al. 2015). As outgroups, we included *H.davidii* Franch., *H.indochinensis* Merr., and *H.febrifuga* (Lour.) Y. De Smet & Granados (*Dichroafebrifuga* Lour.) collected in Vietnam (Table 1), where we carried out a series of field studies (Middleton et al. 2019; Nagahama et al. 2021). In each sample, a small leaf piece was cut out, placed in a tea bag, and dried with silica gel in a zip-lock bag.

**Table 1.** Samples used in molecular phylogenetic analyses.

Scientific name	Voucher ID	Locality	Coordinates
Hydrangea (Dichroa) sp.	V8372	Bidoup Nui Ba, Vietnam	12.16016944, 108.5364333
*Hydrangeaacuminata* [Shikoku lineage]	TGK0472	Ino, Kochi	33.781458, 133.188252
*Hydrangeaacuminata* [Shikoku lineage]	JPN3301	cultivated, Fukuoka	33.55545001, 130.1939861
Hydrangeaacuminatassp.acuminata	JPN0330	Mt. Ihara, Fukuoka	33.48363400, 130.2638410
Hydrangeaacuminatassp.acuminata	JPN0433	Mt. Raizan, Fukuoka	33.48293333, 130.2204444
Hydrangeaacuminatassp.acuminata	JPN2336	Mt. Oyaji, Miyazaki	32.77326944, 131.3367306
Hydrangeaacuminatassp.acuminata	JPN2063	Mt. Shiraiwa, Miyazaki	32.56233100, 131.1113540
Hydrangeaacuminatassp.australis	JPN0908	Mt. Karakuni, Miyazaki	31.93438888, 130.8600000
Hydrangeaacuminatassp.australis	JPN3192	Miyakonojyo, Miyazaki	31.78877222, 130.9603278
Hydrangeaacuminatassp.yakushimemsis	JPN1708	Yakushima, Kagoshima	30.372031, 130.504266
Hydrangeaacuminatassp.yakushimemsis	JPN1799	Yakushima, Kagoshima	30.34255555, 130.4810000
*Hydrangeadavidii*	V4997	Fansipan, Vietnam	22.34225, 103.7764167
*Hydrangeagrosseserrata*	JPN0528	Yakushima, Kagoshima	30.34619444, 130.3918750
*Hydrangeagrosseserrata*	JPN0652	Yakushima, Kagoshima	30.26264444, 130.5800944
*Hydrangeahirta*	JPN2415	Mt. Amagi, Shizuoka	34.86201944, 139.0215139
*Hydrangeaindochinensis*	V4959	Fansipan, Vietnam	22.34755555, 103.7721944
*Hydrangeakawagoeana*	TG00879	Suwanose-jima, Kagoshima	29.62290600, 129.69778900
*Hydrangealuteovenosa*	JPN0378	Mt. Ihara, Fukuoka	33.48294444, 130.2541972
*Hydrangealuteovenosa*	JPN0901	Mt. Karakuni, Miyazaki	31.93438888, 130.8600000
*Hydrangealuteovenosa*	JPN1982	Mt. Osuzu, Miyazaki	32.29758800, 131.4459520
*Hydrangeamacrophylla*	JPN3302	cultivated, Fukuoka	33.55545001, 130.1939861
*Hydrangeamacrophylla*	JPN3303	cultivated, Fukuoka	33.55545001, 130.1939861
*Hydrangeaminamitanii*	JPN1983	Mt. Osuzu, Miyazaki	32.29758800, 131.4459520
*Hydrangeaminamitanii*	TG01200	Aya, Miyazaki	32.03053900, 131.21502800
*Hydrangeascandens*	JPN1980	Mt. Osuzu, Miyazaki	32.29758800, 131.4459520
*Hydrangeascandens*	JPN2931	Kihoku, Mie	34.18644999, 136.1858528
Hydrangeaserratavar.angustata	JPN2404	Izu City, Shizuoka	34.96862800, 138.8459450
Hydrangeaserratavar.serrata	JPN2980	Osugi-dani, Mie	34.21346388, 136.1650250

### ﻿DNA isolation, genome-wide Single Nucleotide Polymorphism (SNP) genotyping, and construction of phylogenetic trees

Total DNA was extracted from the dried leaves using the cetyl trimethyl ammonium bromide (CTAB) method (Doyle and Doyle 1990). Multiplex ISSR genotyping by sequencing (MIG-seq, Suyama and Matsuki 2015) was used for *de novo*SNP detection. Briefly, a MIG-seq library was prepared by a two-step PCR amplification process based on the protocol detailed by Suyama et al. (2022). The amplicons in the size range of 300–800 bp were purified and sequenced on an Illumina MiSeq platform (Illumina, San Diego, CA, USA) using an MiSeq Reagent Kit v3 (150 cycles, Illumina). We skipped the sequencing of the first 17 bases of reads 1 and 2 (SSR primer regions and anchors) using “DarkCycle”. Low-quality reads and extremely short reads containing adapter sequences were removed using Trimmomatic 0.39 (Bolger et al. 2014). Stacks 2.41 pipeline software (Catchen et al. 2013; Rochette et al. 2019) was used to obtain individual genotypes with the following parameters: minimum depth of coverage required to create a stack (*m*) = 3, maximum distance between stacks (*M*) = 2, maximum mismatches between loci when building the catalog (*n*) = 2. Three different filtering criteria were applied for quality control of the SNP data. First, any SNP site where one of two alleles had less than three counts was filtered out because it is difficult to distinguish polymorphisms from sequencing errors when the minor allele count of SNPs is too low (Roesti et al. 2012). Second, loci containing SNPs with high heterozygosity (Ho ≥ 0.6) were removed because excess heterozygosity may have resulted from artifactual loci built from several paralogous genomic regions. Third, SNPs with a genotyping rate of < 50% were eliminated. Using the third criterion, the SNPs that were retained by 14 or more samples remained in the SNP dataset.

Maximum likelihood phylogeny based on SNPs was inferred using software RAxML 8.2.10 (Stamatakis 2014). We used a GTRCAT model and performed 1,000 replicates of parallelized tree search bootstrapping. Based on the clades of the MIG-seq tree, we estimated pairwise *F*_ST_ values for each clade using the POPULATIONS program in Stacks.

### ﻿Sequencing and phylogenetic analysis of chloroplast and nuclear genomic regions

The chloroplast and nuclear genomic regions were sequenced using the next generation sequencing (NGS) technique (Suyama et al. 2022). First, three chloroplast genomic regions, *rbc*L, *trn*L intron, and *psb*A-*trn*H, and two nuclear genomic regions, ITS1 and ITS2, were simultaneously amplified using the Multiplex PCR Assay Kit Ver. 2 (Takara Bio, Kusatsu, Japan) (first PCR reaction). The first primers consisted of tail sequences and locus-specific primers (Suyama et al. 2022). Second, the products from the first PCR reaction were purified and used for the second PCR. The second PCR was conducted using primer pairs including tail sequences, adapter sequences for Illumina sequencing, and the index sequence to identify each individual sample. Third, the second PCR products from each sample were mixed, and sequencing was performed using an Illumina MiSeq platform with an MiSeq Reagent Nano Kit v2 (500 cycles, Illumina). We skipped the sequencing of the first three bases of reads 1 and 2 (anchor region for the 2^nd^ PCR primer) using the “DarkCycle” option of the MiSeq system. Both ends of the fragments and index sequences were read by paired-end sequences (reads 1 and 2) and index sequencing. The number of bases read was 251 bases for both read 1 and read 2.

The sequences of the five regions were determined using Claident pipeline (Tanabe and Toju 2013, http://www.claident.org/, Tanabe, A.S., Claident, Date of access: 05/01/2021). First, raw MiSeq BCL data were converted into FASTQ data using the BCL2FASTQ program provided by Illumina, and raw FASTQ data were demultiplexed based on index and primer sequences using the clsplitseq program in Claident. Subsequent analysis was performed per region per individual. In ITS1 and ITS2, we merged paired-end reads because reads 1 and 2 overlapped. In *rbc*L, *trn*L intron, and *psb*A-*trn*H, we independently analyzed reads 1 and 2 because the length of the sequenced reads was too short to merge reads 1 and 2. Second, the low-quality 3’ tails were trimmed and the low-quality sequences were filtered out using the clfilterseq program. Third, the noisy and chimeric sequences were removed using the clcleanseqv program. Fourth, the remaining reads were clustered with a cut-off sequence similarity of 99%. An operational taxonomic unit (OUT) that had the most observed reads within the individual was treated as a representative OTU sequence.

Multiple alignments of the chloroplast and nuclear genomic regions were performed using the program MAFFT 7.313 (Katoh and Standley 2013), and alignment columns containing gaps were trimmed using a heuristic selection method based on similarity statistics of trimAl 1.4.rev15 (Capella-Gutiérrez et al. 2009). We used Kakusan 4.0 (Tanabe 2011) to find suitable nucleotide substitution models and partitioning strategies for the nucleotide datasets. The chloroplast and nuclear genomic regions were independently run through Kakusan. The corrected Akaike Information Criterion (AICc; Sugiura 1978) was used to compare nonpartitioned, partitioned _ equal _ mean _ rate, and separate models. The nonpartitioned model (GTR + Γ) proved optimal for both the chloroplast and nuclear genomic regions. Maximum likelihood phylogenies were inferred using RAxML 8.2.10 (Stamatakis 2014), whereby 1,000 replicates of parallelized tree search bootstrapping were conducted.

### ﻿Morphological observations

Using the specimens listed in Table 2, we measured the following leaf traits using the largest leaf: leaf blade length, leaf blade width, petiole length, leaf apex length, leaf teeth length, and the number of teeth on one side of the leaf margin. Leaf teeth length was measured as the height from the line between two bases of a tooth to the tip of the tooth, for the highest tooth of the largest leaf. We also measured the corymb length, corymb width, and capsule length for fruiting specimens.

**Table 2.** The specimens used for measurements of nine morphological traits.

Taxa	Specimen ID	Herbaria
Hydrangeaacuminatassp.acuminata	KAG161334, KAG161335, KAG161336, KAG161337, KAG161338, KAG161344, KAG161345, KAG161348, KAG161349, KAG161350	KAG
Hydrangeaacuminatassp.acuminata	Fujii 117037	KYO
Hydrangeaacuminatassp.australis	KAG023305, KAG083840, KAG083882, KAG086731, KAG161312, KAG161315, KAG161327, KAG161377	KAG
Hydrangeaacuminatassp.australis	Fujii 18200, Fujii 178001	KYO
Hydrangeaacuminatassp.yakushimensis	Yahara et al. 791, 792, 793–1, 793–2, 793–3, 793–4, 1103, 1104, 1105, JPN1799	FU

### ﻿Data resources

All raw MIG-seq data were deposited at the DDBJ Sequence Read Archive (DRA) with accession number DRA011509. The demultiplexed raw reads of ITS and cpDNA regions were deposited at the DDBJ Sequence Read Archive (DRA) with accession number DRA011510. All sequences of ITS and cpDNA regions were registered to DNA Data Bank of Japan (DDBJ) under accession nos. LC657594–LC657817.

## ﻿Results

### ﻿Phylogenetic and population genetic analyses using MIG-seq

A total of 22,106,838 raw reads (789,530 ± 47,627 reads per sample) were obtained, and after quality control, 20,944,147 reads (748,005 ± 45,296 reads per sample) remained. After *de novo*SNP detection and filtering, the dataset had 1,746 SNPs from 685 loci.

**Figure 2.**
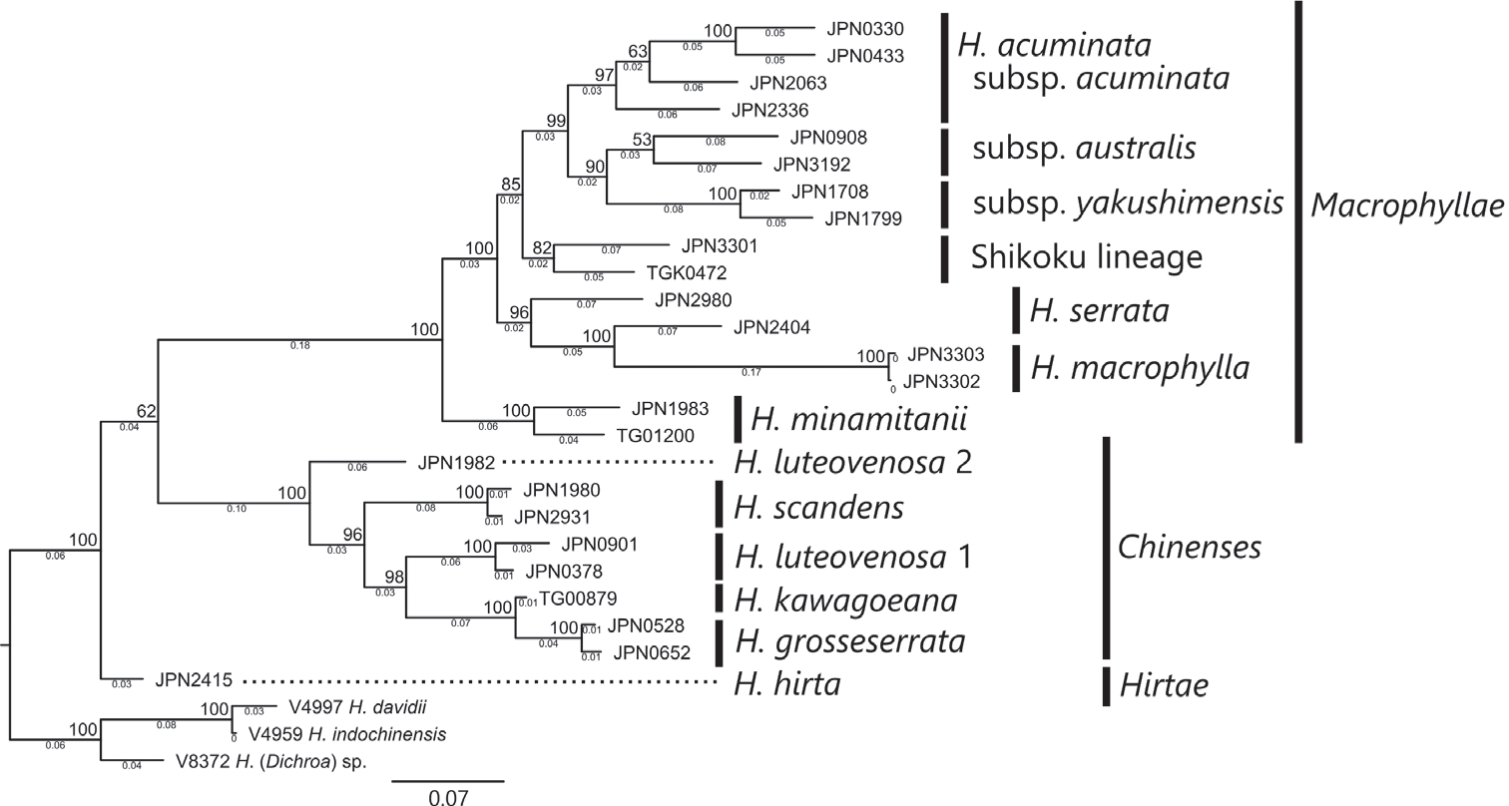
Molecular phylogenetic tree reconstructed using MIG-seq. Bootstrap values are shown on the nodes, and branch lengths are shown on the internodes. Branch length represents the average number of substitutions per SNP site.

In the MIG-seq tree (Fig. 2), nine *Hydrangea* species were clustered into three clades corresponding to sect.Macrophyllae (*H.acuminata*, *H.macrophylla*, *H.minamitanii*, and *H.serrata*), sect.Chinenses (*H.grosseserrata*, *H.kawagoeana*, *H.luteovenosa*, and *H.scandens*), and sect.Hirtae (*H.hirta*). In the *Macrophyllae* clade, *H.minamitanii* was sister to the clade including the other three species and monophylies of both *H.minamitanii* and the latter clade were supported by 100% bootstrap values. Among the latter three species, the clade including *H.macrophylla* and *H.serrata* was supported by a 96% bootstrap value and sister to the clade of *H.acuminata* supported by an 85% bootstrap value. Within *H.acuminata*, the Shikoku lineage was sister to a clade supported by a 99% bootstrap value including subsp.acuminata, subsp.australis, and subsp.yakushimensis, and the sister relationship of subsp.australis and subsp.yakushimensis was supported by a 90% bootstrap value. Even after the separation of *H.acuminata*, *H.serrata* was not monophyletic. The samples of *H.serrata* from Mie (JPN2980; var.serrata) and Shizuoka (JPN2404; var.angulata) were clustered with *H.macrophylla* but not sister to each other, and the sister relationship of H.serratavar.angulata and *H.macrophylla* was supported by a 100% bootstrap value. Similarly, *H.luteovenosa* was not monophyletic. Whereas *H.luteovenosa* 1 was sister to a clade including *H.kawagoeana* and *H.grosseserrata*, *H.luteovenosa* 2 was sister to a clade including all the other samples of sect.Chinenses.

The degree of genetic differentiation measured by *F_ST_* (Table 3) was 0.251 between H.acuminatasubsp.acuminata and subsp.australis , 0.316 between subsp.acuminata and subsp.yakushimensis , and 0.437 between subsp.australis and subsp.yakushimensis. Among the closely related species of sect.Macrophyllae, *F_ST_* was 0.553 between *H.macrophylla* and *H.serrata*, 0.317–0.514 between *H.acuminata* and *H.serrata*, and 0.452–0.652 between *H.acuminata* and *H.macrophylla*. *Hydrangeaminamitanii* is differentiated from H.acuminatasubsp.acuminata , subsp.australis , subsp.yakushimensis, Shikoku lineage, *H.serrata*, and *H.macrophylla* in *F_ST_* values of 0.340, 0.470, 0.546, 0.439, 0.480, and 0.657, respectively. Between species of sect.Chinenses, *F_ST_* varied from 0.395 (*H.kawagoeana* vs. *H.grosseserrata*) to 0.632 (*H.grosseserrata* vs. *H.scandens*). Between sections, *F_ST_* varied from 0.454 (*H.luteovenosa* 2 of sect.Chinenses vs. H.acuminatasubsp.acuminata) to 0.814 (*H.hirta* vs. *H.macrophylla*).

**Table 3.** The degrees of genetic differentiation between taxa measured by *F_ST_*.

		*Hirtae*	*Chinenses*				*Macrophyllae*						
		*H.grosseserrata*	*H.kawagoeana*	*H.luteovenosa* 1	*H.scandens*	*H.luteovenosa* 2	*H.minamitanii*	H.acuminatassp.acuminata	H.acuminatassp.australis	H.acuminatassp.yakushimensis	*Shikoku lineage*	*H.serrata*	*H.macrophylla*
*Hirtae*	*H.hirta*	0.739	0.696	0.700	0.693	0.624	0.724	0.511	0.654	0.720	0.637	0.715	0.814
*Chinenses*	*H.grosseserrata*	–	0.395	0.580	0.632	0.616	0.749	0.590	0.705	0.769	0.711	0.735	0.808
*Chinenses*	*H.kawagoeana*	–	–	0.473	0.561	0.524	0.768	0.578	0.697	0.736	0.695	0.734	0.792
*Chinenses*	*H.luteovenosa* 1	–	–	–	0.544	0.510	0.729	0.574	0.681	0.728	0.687	0.702	0.775
*Chinenses*	*H.scandens*	–	–	–	–	0.551	0.722	0.574	0.684	0.759	0.695	0.711	0.787
*Chinenses*	*H.luteovenosa* 2	–	–	–	–	–	0.501	0.454	0.594	0.679	0.565	0.598	0.720
*Macrophyllae*	*H.minamitanii*	–	–	–	–	–	–	0.340	0.470	0.546	0.439	0.480	0.657
*Macrophyllae*	H.acuminatassp.acuminata	–	–	–	–	–	–	–	0.251	0.316	0.257	0.317	0.452
*Macrophyllae*	H.acuminatassp.australis	–	–	–	–	–	–	–	–	0.437	0.405	0.441	0.606
*Macrophyllae*	H.acuminatassp.yakushimensis	–	–	–	–	–	–	–	–	–	0.453	0.514	0.652
*Macrophyllae*	Shikoku lineage	–	–	–	–	–	–	–	–	–	–	0.364	0.585
*Macrophyllae*	*H.serrata*	–	–	–	–	–	–	–	–	–	–	–	0.553

### ﻿Phylogenetic tree reconstructed using ITS sequences

**Figure 3.**
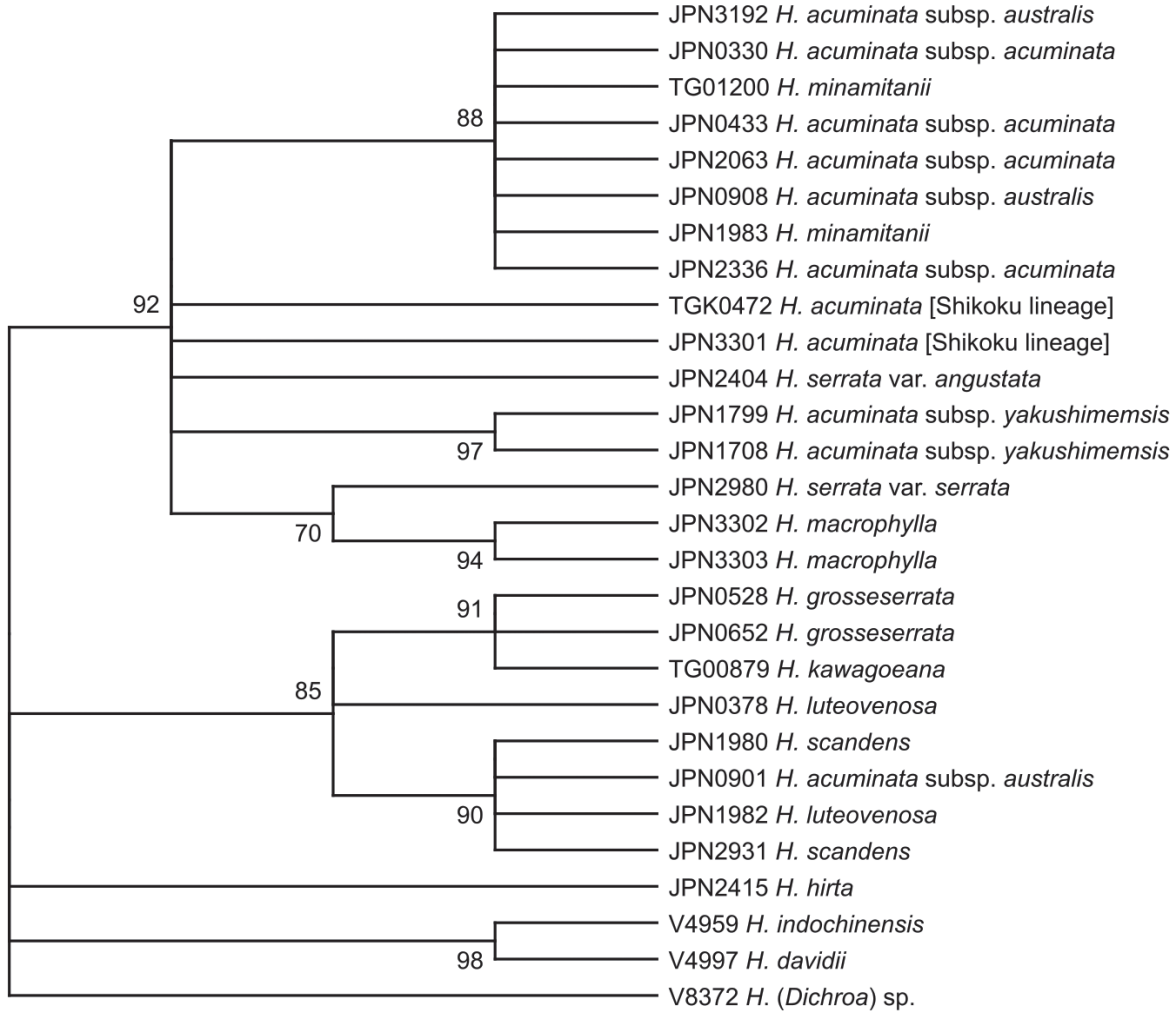
Molecular phylogenetic tree reconstructed using ITS sequences. Bootstrap values are shown on the nodes. Nodes supported by less than 70% bootstrap values are not shown.

A total of 111,216 reads (3,972 ± 299 reads per sample, ITS1) and 81,988 reads (2,928 ± 155 reads per sample, ITS2) were obtained. After gaps were trimmed, the total length of the sequences was 635 bp (ITS1: 267 bp, ITS2: 368 bp). In the ITS tree (Fig. 3), sect.Macrophyllae was supported by a 92% bootstrap value, and sect.Chinenses was supported by an 85% bootstrap value. In sect.Macrophyllae, only three branches were supported by bootstrap values larger than 80%: a clade including *H.acuminata* and *H.minamitanii* was supported by an 88% bootstrap support, H.acuminatasubsp.yakushimensis was supported by 97%, and *H.macrophylla* was supported by 94%. In sect.Chinenses, a clade including *H.kawagoeana* and *H.grosseserrata* was supported by a 91% bootstrap value, and another clade including *H.luteovenosa* 1 and *H.scandens* was supported by a 90% bootstrap value. *Hydrangealuteovenosa* 1 and *H.luteovenosa* 2 were not sister to each other.

### ﻿Phylogenetic tree reconstructed using cpDNA sequences

**Figure 4.**
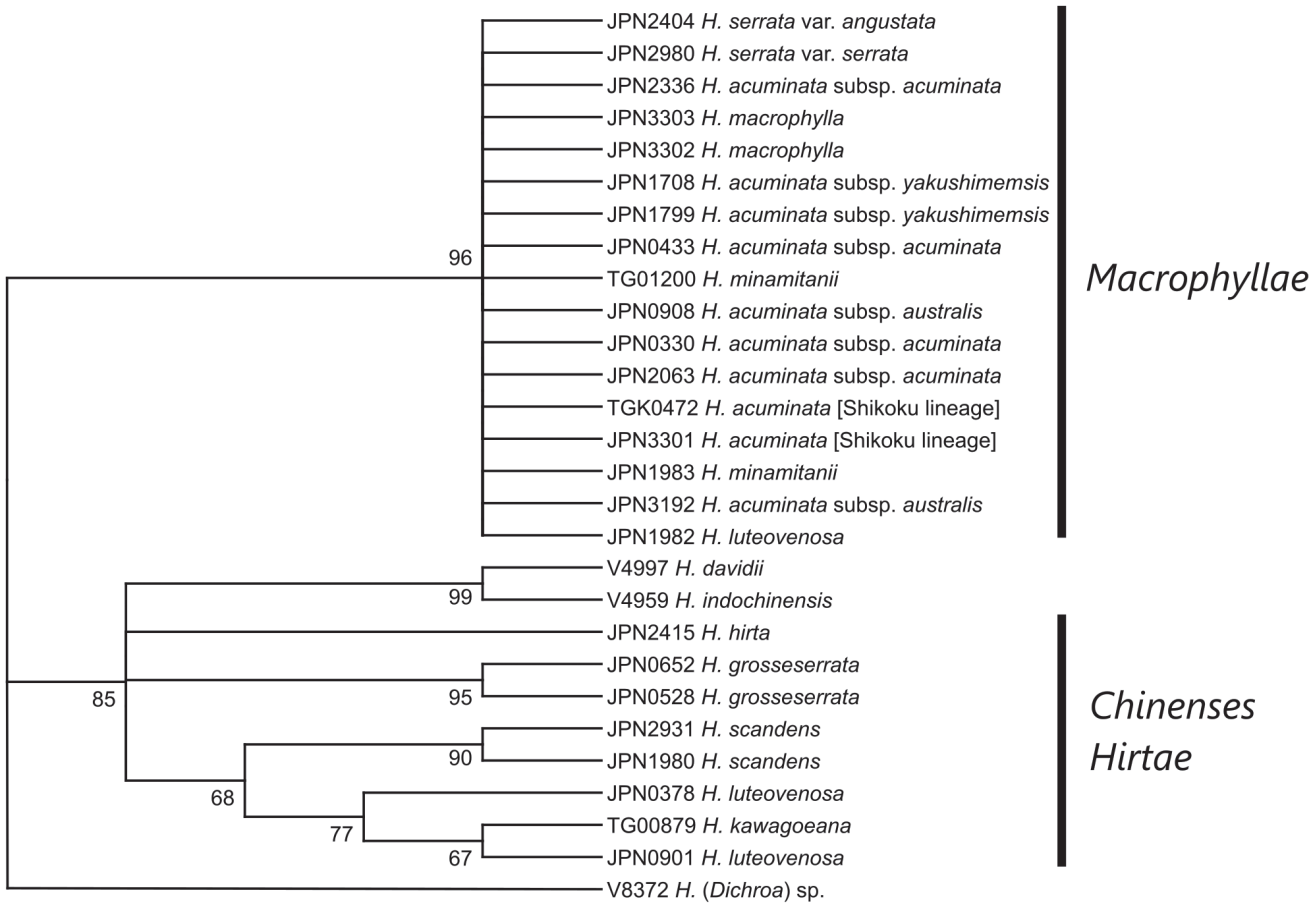
Molecular phylogenetic tree reconstructed using cpDNA sequences. Bootstrap values are shown on the nodes. Nodes supported by less than 60% bootstrap values are not shown.

A total of 20,290 reads (725 ± 68 reads per sample, *rbc*L), 18,724 reads (669 ± 68 reads per sample, *trn*L intron), and 20,194 reads (721 ± 72 reads per sample, *psb*A-*trn*H) were obtained. After gaps were trimmed, the total length of the sequences was 1,354 bp. The sequenced lengths of each region were 222 bp and 227 bp (read 1 and 2 of *rbc*L), 228 bp and 228 bp (read 1 and 2 of *trn*L intron), and 226 bp and 223 bp (read 1 and 2 of *psb*A-*trn*H). In the cpDNA tree reconstructed using these sequences (Fig. 4), the monophyly of sect.Macrophyllae was supported by a 96% bootstrap value, and the two lineages of sect.Chinenses and *H.hirta* were polychotomous.

### ﻿Morphological observations

**Figure 5.**
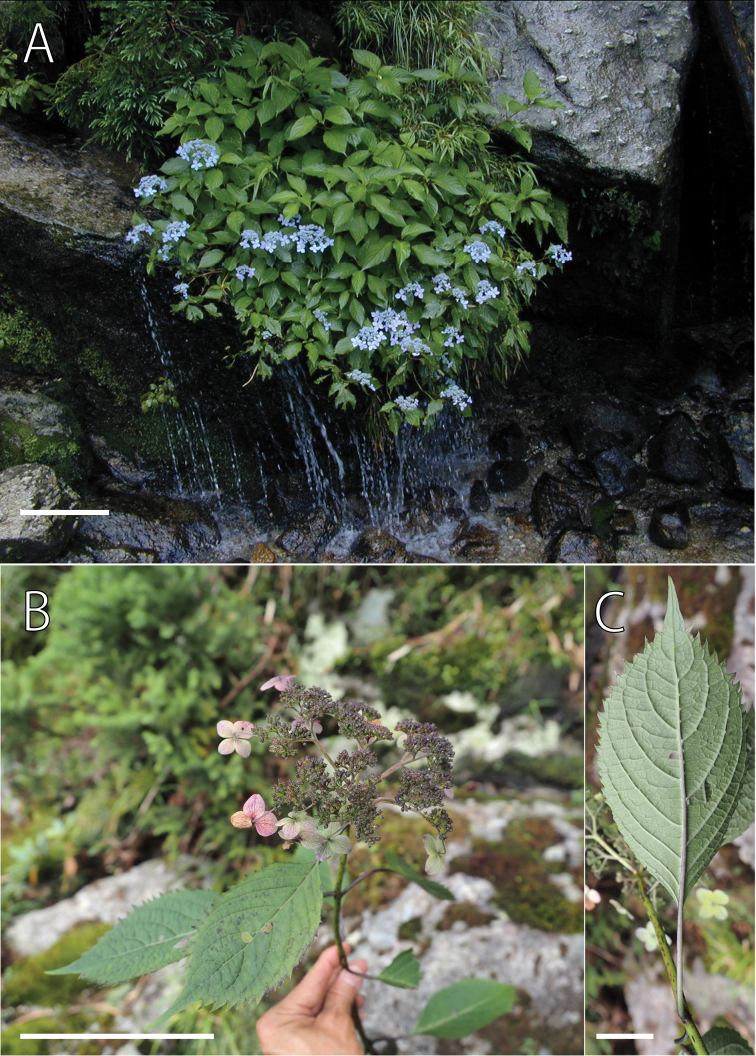
Hydrangeaacuminatasubsp.yakushimensis Yahara & Tagane **A** a tree growing on cliff along stream **B** a fruiting twig of the specimen *JPN1799* (holotype) **C** lower leaf surface of the specimen *JPN1799*. Scale bars: 20 cm (**A**); 10 cm (**B**); 2 cm (**C**).

**Figure 6.**
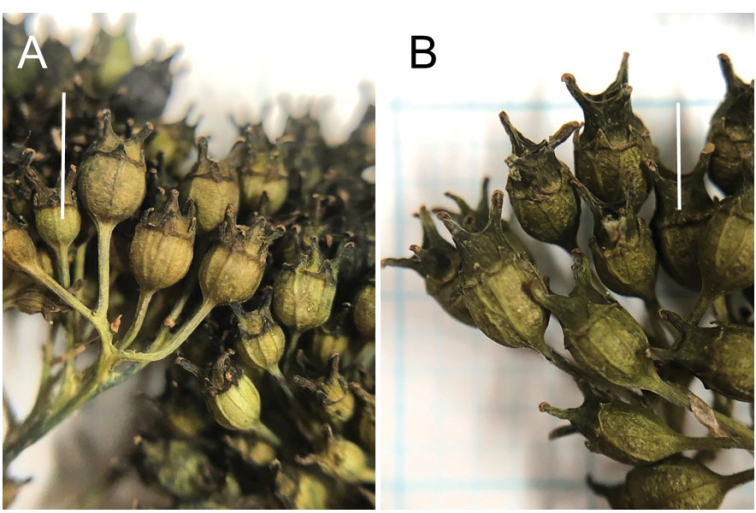
Fruits of Hydrangeaacuminatasubsp.yakushimensis Yahara & Tagane **A** and subsp. acuminata**B** Specimen: *JPN1799* (holotype) **A***JPN2063***B**. Scale bars: 3 mm.

Morphologically, H.acuminatasubsp.yakushimensis is similar to subsp.acuminata in having blue-colored flowers: fertile flowers with blue-colored petals, stamens, and sterile flowers with blue-colored calyces (Fig. 5). However, H.acuminatasubsp.yakushimensis is distinct from subsp.acuminata in that the upper leaf surface is glabrous except on veins (vs. sparsely hairy), the lower leaf surface is glabrous or only slightly hairy except for tufted hairs at axils of lateral veins (vs. sparsely hairy), and capsules are shorter than 2.7 mm (vs. 3.2 mm or longer; Fig. 6; Table 4; Table 5). In addition, H.acuminatasubsp.yakushimensis is different from subsp.acuminata in that the number of teeth along each margin of the largest leaf exceeds 27 (vs. 27 or fewer in subsp.acuminata), the length of leaf serrations of the largest leaf exceeds 3 mm (vs. 1.0–2.9 mm), and the width of infructescence attains to 7–12 cm (vs. 3.2–8.7 cm).

**Table 4.** Measurements for nine morphological traits of H.acuminatassp.acuminata , ssp.australis , ssp.yakushimensis, and *H.minamitanii*.

	H.a.ssp.yakushimensis	H.a.ssp.acuminata	H.a.ssp.australis	*H.minamitanii*
Leaf length	12.5±1.5 (10.2–14.4) cm	10.4±2.8 (10.4–15.6) cm	13.4±1.6 (10.1–15.4) cm	12.6±1.1 (12–14) cm
Leaf width	6.2±0.9 (4.6–7.5) cm	4.3±1.1 (2.8–6.2) cm	7.7±1.4 (5.4–9.7) cm	6.2±0.9 (5.0–6.9) cm
Petiole length	2.8±1.0 (1.2–5.3) cm	2.1±0.8 (0.9–3.5) cm	2.8±1.3 (0.9–5.0) cm	3.0±1.2 (2.2–4.5) cm
Leaf apex length	1.7±0.5 (1.0–2.4) cm	1.8±0.7 (0.9–2.9) cm	1.7±0.5 (0.7–2.5) cm	1.5±0.5 (0.8–1.9) cm
Leaf teeth length	2.8±1.1 (1.0–5.0) mm	1.8±0.6 (1.0–2.9) mm	3.0±1.0 (1.0–5.0) mm	3.1± 0.8 (2.2–4.2) cm
No of teeth	28.5±7.1 (15–42)	21.2±6.1 (9–27)	28.5±7.1 (15–42)	28.8±4.9 (23–35)
Corymb length	4.3±1.5 (2.0–7.0) cm	4.1±1.5 (1.9–6.3) cm	5.8±2.2 (3.0–9.2) cm	5.1±0.9 (4.5–6.3) cm
Corymb width	6.7±2.7 (3.9–11.8) cm	5.5±1.9 (3.2–8.7) cm	8.9±2.5 (6.2–12.8) cm	7.9±0.9 (7.2–9.2) cm
Capsule length	2.4±0.2 (2.2–2.7) mm	3.9±0.6 (3.2–5.1) mm	4.3±0.4 (3.8–4.9) mm	4.6±1.0 (3.9–5.3) mm

Phylogenetically, H.acuminatasubsp.yakushimensis is sister to subsp.australis. Morphologically, H.acuminatasubsp.yakushimensis is similar to subsp.australis in having leaves larger than subsp.acuminata but distinguished with leaves glabrous adaxially except veins (vs. sparsely hairy in subsp.australis; Table 5) and capsule less than 3 mm long (Table 4).

**Table 5.** Morphological comparison between H.acuminatassp.acuminata , ssp.australis , ssp.yakushimensis, and *H.minamitanii.*

	H.a.ssp.yakushimensis	H.a.ssp.acuminata	H.a.ssp.australis	*H.minamitanii*
Upper surface of lamina	glabrous	sparsely hairy	sparsely hairy	glabrous
Upper surface of veins	hairy	hairy	hairy	hairy
Lower surface of lamina	glabrous	sparsely hairy	densely curled hairy	glabrous
Lower survace of veins	glabrous or glabrescent	sparsely hairy	densely curled hairy	glabrous or glabrescent
Axils of lateral veins	hairs densely tufted	hairs not densely tufted	hairs not densely tufted	hairs densely tufted
Petiole	glabrous	hairy	densely hairy	glabrous
Young shoot	glabrous	hairy	densely hairy	glabrous
Calyx of showy flower	blue	blue	blue	pink or white

## ﻿Discussion

The discovery of H.acuminatasubsp.yakushimensis is surprising because Yakushima is a well-botanized island, and H.acuminatasubsp.yakushimensis has conspicuous, blue-colored flowers. This discovery illustrates that botanical surveys in the mountain-top area of Yakushima still remain insufficient, most likely because of its steep topography. In fact, our recent surveys resulted in the discovery of not only H.acuminatasubsp.yakushimensis but also an additional new taxon of *Stellaria* (Caryophyllaceae) (Yahara et al. 2021b). Further field surveys including researchers who have more experience climbing mountains and steep cliffs could result in the discovery of even more undescribed taxa from the mountain-top area of Yakushima.

Using RAPD and the sequences of *rbc*L and *mat*K, Uemachi et al. (2014) showed that H.serratavar.serrata s. lat. diverged to western and eastern groups, corresponding to *H.acuminata* and H.serratavar.serrata s. str., respectively. However, Uemachi et al. (2014) did not examine H.acuminatasubsp.australis, H.acuminatasubsp.yakushimensis, and *H.minamitanii*. The MIG-seq tree (Fig. 2) revealed that *H.minamitanii* is sister to the clade including *H.acuminata*, *H.serrata*, and *H.macrophylla. Hydrangeaminamitanii* is differentiated from the other species of sect.Macrophyllae with *F_ST_* values from 0.340 to 0.657, and this difference was equivalent to the *F_ST_* variation between the species of sect.Chinenses from 0.395 (*H.kawagoeana* vs. *H.grosseserrata*) to 0.632 (*H.grosseserrata* vs. *H.scandens*). These findings support the treatment of *H.minamitanii* as a distinct species.

In contrast, the *F_ST_* between H.acuminatasubsp.acuminata and subsp.australis (0.251) is lower than the above values (0.340 to 0.657) observed between the species, supporting the treatment as two subspecies. Similarly, the *F_ST_* between H.acuminatasubsp.acuminata and subsp.yakushimensis was 0.316, which is considered to be at the subspecies level, and the *F_ST_* between subsp.australis and subsp.yakushimensis (0.437) was slightly higher. Differences between H.acuminatasubsp.acuminata and subsp.australis are smaller, not only genetically, but also morphologically: JPN0908 collected at 1700-m elevation on Mt. Karakuni was identified as subsp.australis in the MIG-seq tree, but is morphologically very similar to subsp.acuminata, suggesting hybridization or intergradation between subsp.acuminata and subsp.australis.

In the MIG-seq tree (Fig. 2), H.acuminatasubsp.yakushimensis was sister to H.acuminatasubsp.australis distributed in the southern part of Kyushu mainland (Kagoshima Pref. and the southern part of Miyazaki Pref.). There are other cases where the endemic plants of Yakushima have related taxa in southern Kyushu. For example, *Asarum* (Araceae, Okuyama et al. 2020), *Mitella* (Saxifragaceae, Okuyama et al. 2005), and *Rhododendron* (Ericaceae, Minamitani et al. 2018) have all been reported as showing this pattern. The sister relationship between H.acuminatasubsp.yakushimensis and subsp.australis provided another case which supported the phytogeographical similarity between the endemic flora of Yakushima and the flora of the southern Kyushu mainland.

The MIG-seq tree (Fig. 2) showed that the Shikoku lineage was distinct from a clade including three subspecies of *H.acuminata* distributed in Kyushu. This finding agrees with the results of Uemachi et al. (2014), showing that the samples from Shikoku were distinct for both *rbc*L and *mat*K sequences from other “western subgroups” corresponding to *H.acuminata*. We did not find differences in *rbc*L sequences between the Shikoku lineage and other samples of *H.acuminata*, which is most likely because we determined shorter sequences of *rbc*L than did Uemachi et al. (2014): 449 bp. vs 1257 bp. The MIG-seq tree and the results described by Uemachi et al. (2014) suggest that the Shikoku lineage may be treated as a fourth subspecies of *H.acuminata*. However, further morphological and molecular phylogenetic studies, using more samples from Shikoku, are needed to conclude the taxonomic treatment of the Shikoku lineage.

The MIG-seq tree (Fig. 2) also showed that *H.serrata* was not monophyletic if *H.macrophylla* was separated as a species; the sample of H.serratavar.angulata was sister to *H.macrophylla*, and the sample of H.serratavar.serrata was basal to this sister pair. This result suggests that *H.serrata* includes multiple species even after *H.acuminata* and *H.minamitanii* are separated. *Hydrangeaserrata* s. lat. is widely distributed from Kyushu to Hokkaido, the northern-most island of Japan. Our samples were limited to the area of western Japan on the Pacific side and did not include H.serratavar.yezoensis. Further studies of populations in central and northern Japan, including more samples of H.serratavar.angulata , var.serrata , and var.yezoensis, are needed to revise the taxonomy of the complex that has been treated as *H.serrata* s. lat.

It is notable that *H.luteovenosa* 1 and *H.luteovenosa* 2 were not sister to each other in both MIG-seq and ITS trees. In the MIG-seq tree which has a higher resolution than the ITS tree, *H.luteovenosa* 2 (JPN1982 collected from Mt. Osuzu, Miyazaki Pref.) was basal to a clade including *H.scandens*, *H.luteovenosa* 1 (JPN0378 collected from Mt. Ihara, Fukuoka Pref.), *H.kawagoeana*, and *H.grosseserrata*. It is likely that *H.luteovenosa* contains two cryptic species. To test this possibility, further studies with more samples of *H.luteovenosa* are needed.

This study demonstrated the usefulness of MIG-seq to obtain finely resolved phylogenetic trees for closely related species and infraspecific taxa in taxonomically complicated groups such as *Hydrangea*. Compared with the ITS and cpDNA trees, where only a few branches were supported by bootstrap values larger than 90%, most branches in the MIG-seq tree were supported by bootstrap values larger than 90%. In the ITS tree, the monophyly of H.acuminatasubsp.yakushimensis was supported by the 97% bootstrap value, but the monophyly of H.acuminatasubsp.acuminata and subsp.yakushimensis was ambiguous; the cluster of H.acuminatasubsp.acuminata and *H.minamitanii* with the bootstap value 88% was weakly consistent with the MIG-seq tree topology. The resolution of the MIG-seq tree is even higher than that of the RAPD tree for the *H.serrata* complex obtained by Uemachi et al. (2014). Other recent studies using MIG-seq on *Hosta* (Yahara et al. 2021a) and *Stellaria* (Yahara et al. 2021b) have also demonstrated its usefulness in resolving taxonomic complexity and describing new taxa. As this method is more applicable to a small number of poor-quality samples than RAD-seq (Binh et al. 2018; Strijk et al. 2020; Zhang et al. 2020), it is expected to be used for taxonomic studies of many groups for which reliable phylogenetic relationships could not be reconstructed by conventional molecular phylogenetic methods.

### ﻿Key to related species

**Table:** 

1	Calyces of marginal showy flowers, petals of fertile flowers, and stamens always pink or white	**2**
–	Calyces of marginal showy flowers, petals of fertile flowers, and stamens light blue when flowering	**3**
2	Leaves glabrous adaxially except veins and glabrous abaxially except for tufted hairs at axils of lateral veins. Distributed in Kyushu	***H.minamitanii***
–	Leaves more or less hairy adaxially and abaxially. Distributed in Honshu	***H.serrata***
3	Leaves glabrous adaxially except veins. Capsules 2.7 mm or shorter	**H.acuminatasubsp.yakushimensis**
–	Leaves hairy adaxially. Capsules 3.2 mm or longer	**4**
4	Leaves usually sparsely hairy abaxially, hair not curled. Leaf width less than 6.2 cm	**H.acuminatasubsp.acuminata**
–	Leaves usually densely hairy abaxially, hair curled. Leaf width often 6.2 cm or larger	**H.acuminatasubsp.australis**

## ﻿Taxonomy

### ﻿ Hydrangea acuminata

Taxon classificationPlantaeCornalesHydrangeaceae

411BBEFB-9EE2-5E8C-B77B-4BE91785EDD3

Hydrangea acuminata Siebold & Zucc., Fl. Jap. 1: 110, t. 56, 57-I (1839); Ohba & Akiyama in Bull. Natl. Mus. Nat. Sci., Ser. B, 39: 178 (2013).

#### Type.

Japan, Higo Province, Kyushu (*L0043373*, the lectotype designated by Ohba and Akiyama (2013)).

### ﻿ Hydrangea acuminata subsp. acuminata

Taxon classificationPlantaeCornalesHydrangeaceae

BCC0D246-40B7-5EC8-97ED-65667967F831

Hortensia serrata var. acuminata (Siebold & Zucc.) H. Ohba & S. Akiyama, J. Jap. Bot. 91: 347 (2016).
Hydrangea macrophylla (Thunb.) Ser. var. acuminata (Siebold & Zucc.) Makino, Ill. Fl. Nippon: 484, f. 1451 (1940), nom. tant.

#### Japanese name.

Sawa-ajisai, Nishino-yama-ajisai.

#### Distribution and habitats.

Hydrangeaacuminatasubsp.acuminata is widely distributed on the main island of Kyushu, and usually grows on the soil near streams and often on cliffs, and sometimes in disturbed habitats.

#### Note.

Ohba and Akiyama (2016) treated this species as a variety of *Hortensiaserrata*. However, our phylogenetic analysis described below supports the treatment of it as a distinct species.

### ﻿ Hydrangea acuminata subsp. yakushimensis

Taxon classificationPlantaeCornalesHydrangeaceae

Yahara & Tagane subsp. nov.

50E676C1-6C27-5E8E-A006-CCED6D6C685B

urn:lsid:ipni.org:names:77248597-1

Figures 4 , 5

#### Diagnosis.

Hydrangeaacuminatasubsp.yakushimensis is different from subsp.acuminata in that it has smaller capsules, 2.2–2.7 mm long with calyx tube 1.2–1.4 mm long and projected apical part including persistent style 1.0–1.3 mm (vs. capsules 3.2–5.1 mm long with calyx tube 1.6–3.4 mm and projected apical part including persistent style 1.5–2.0 mm), a larger infructescence attaining to 7 × 12 cm (vs. attaining to 6.3 × 8.7 cm), leaves glabrous adaxially except veins (vs. hairy) and glabrous or only slightly hairy abaxially except for tufted hairs at axils of lateral veins (vs. hairy overall on abaxial surface).

#### Type.

Japan. Kagoshima Pref.:Yakushima Migitani, on cliff along stream, 30.34255555°N, 130.48100000°E, 1520 m elevation, 9 September 2020, with fruits, *K. Fuse JPN1799* (holotype: KYO!).

#### Description.

Shrubs 1–1.5 m tall. First year’s twigs green when fresh, with dark purple lenticels, glabrous, terete. Old twigs pale brown; bark not peeled off. Leaves opposite; petioles purplish green, 1.7–3 cm long, glabrous; leaf blade adaxially green, abaxially light green when fresh, pale green when dried, elliptic, 9–12 × 4.6–6.4 cm, papery, adaxially glabrous except veins which are covered with minute hairs, abaxially glabrous or only sparsely hairy except for tufted hairs at axils of lateral veins, secondary veins 6–9 on each side of midvein, adaxially slightly sunken, abaxially slightly elevated, base broadly cuneate, apex long acuminate, margin serrate, teeth 2–3 mm high, 13–31 along each side of the margin. Inflorescences corymbose cymes, 2–7 cm long, 4–12 cm in diam., densely pubescent, apex flat to slightly arcuate, 3–5-branched; the longest internode of each branch 1.5–2.5 cm long, densely pubescent; infructescence attaining to 7 × 12 cm. Marginal showy flowers light blue, on pedicel 1–2 cm long; sepals 3 to 5, rhomboid-elliptic, 0.8–1.4 × 0.5–1.1 cm, glabrous, apex obtuse, base rounded to cuneate, margin entire. Fertile flowers protandrous, light blue. Male-stage flowers on pedicel 1–1.8 mm long; calyx tube funnel-shaped, ca. 1 mm long, 0.8 mm in diam., lobes 5, triangular, 0.5 × 0.4 mm, apex acute; petals 5, light blue, elliptic, 2–2.2 × 1 mm, glabrous, apex acute; stamens 10, light blue, subequal, filaments 1.5–3 mm long, glabrous, anthers white, globular, 0.6 mm in diam.; ovary nearly 1/2 superior, style 3, connate at base, slightly spreading, dark blue, ca. 0.7 mm long, stigma flat. In female-stage flowers, petals and stamens fallen off; ovary nearly 1/2 superior; calyx tube light blue, ca. 1 mm long; style darker blue, spreading, ca. 1 mm long; capsules 2.2–2.7 mm long; calyx tube subglobose, 1.2–1.4 mm long, 1.5–2 mm in diam., projected apical part including persistent styles 1.5 mm long. Seeds light brown, elliptic, 0.8 × 0.5 mm, not winged.

#### Japanese name.

Yakushima-ruri-ajisai.

#### Phenology.

Flowers were collected in July and August, and fruits were collected in September.

#### Distribution and habitat.

Yakushima (Yaku Island), Japan (endemic). The distribution of H.acuminatasubsp.yakushimensis is restricted to cliffs along streams at Yakushima. It mainly grows in the mountain-top area from 1520 to 1750 m, but one population occurs at an elevation of 575 m, along the Miyanoura River.

#### Etymology.

The specific epithet is derived from the type locality, Yakushima.

#### IUCN Conservation status.

Endangered (EN) based on criterion D; the population size is above 50, but less than 250.

#### Additional specimens examined.

Japan. Kagoshima Pref., Yakushima: Mt. Nagata, on cliff, 30.343799°N, 130.492056°E, 1750 m elevation, 2 August 2005, with flowers, *T. Yahara*, *S. Tagane*, *K. Fuse & T. Saito 0791* (FU!); Kamisamano-kubo, on cliff, 30.343799°N, 130.492056°E, 1750 m elevation, 2 August 2005, with flowers, *T. Yahara*, *S. Tagane*, *K. Fuse & T. Saito 0792* (FU!); ditto, with flowers, *T. Yahara*, *S. Tagane*, *K. Fuse*, *T. Saito 0793* (FU!); Nemachino-kubo, on cliff, 30.345465°N, 130.49468230°E, 1740 m elevation, 12 July 2006, sterile, *S. Tagane & K. Fuse 1065* (FU!); Migitani, on cliff along stream, 30.34255555°N, 130.48100000°E, 1520 m elevation, 13 July 2006, with flowers, *S. Tagane & K. Fuse 1103*, *1104*, *1105* (FU!); Sensuikyo, 30.372031°N, 130.504266°E, 575 m elevation, 31 August 2020, sterile, *K. Fuse JPN1708* (FU!).

### ﻿ Hydrangea acuminata subsp. australis

Taxon classificationPlantaeCornalesHydrangeaceae

(T. Yamaz.) Yahara stat. nov.

FDC82B88-7F8B-527C-8605-FE9A93FBA366

urn:lsid:ipni.org:names:77248598-1

Hydrangea serrata var. australis T. Yamaz., J. Jap. Bot. 76: 175 (2001). **Type.** Japan. Kagoshima Pref., Mt. Takakuma, 11 August 1942, *T. Yamazaki s.n.* (TI).
Hortensia serrata var. australis (T. Yamaz.) H. Ohba & S. Akiyama in Ohashi et al., Wild Fl. Jap. rev. ed. 4: 166 (2017), comb. nud.

#### Japanese name.

Nangoku-yama-ajisai.

#### Distribution and habitats.

Hydrangeaacuminatasubsp.australis is widely distributed at lower elevations in the Kagoshima Prefecture and the southern part of the Miyazaki Prefecture of the Kyushu Island and usually grows in disturbed places along the margins of evergreen forests or *Cryptomeria* plantations. JPN0908 was collected on a volcanic cliff at 1700 m elevation on Mt. Karakuni and was identified as subsp.australis in the MIG-seq tree (Fig. 2).

#### Note.

Hydrangeaacuminatasubsp.australis is distinguished from subsp.acuminata mainly by its larger and wider leaves often exceeding 6.2 cm wide (vs. not exceeding 6.2 cm), having more serrations along margin (22–43 vs. 9–27) and dense curled hair on the lower surface of lamina. However, JPN0908, was identified as subsp.australis in the MIG-seq tree, which is morphologically similar to subsp.acuminata in having smaller leaves, fewer serrations, and sparser pubescence on the lower surface of the leaf. This specimen might be of hybrid origin between subsp.australis and subsp.acuminata.

#### Representative specimens examined.

Japan. Kagoshima Pref.: Kagoshima City, 22 July 2002, with flowers, *K. Maruno s.n*. (KAG 083840!); Shibushi City, 4 June 2002, with fruits, *K. Maruno s.n*. (KAG 083882!); Aira City, 11 July 2004, *K. Maruno s.n*. (KAG 086731!); Kimotsuki Town, 300 m elevation, 20 July 1986, with fruits, *S. Hatusima 41199* (KAG 161312!); KHydrangeahima City, 450 m elevation, 22 November 1986, with fruits, *S. Hatusima 41920* (KAG 161315!); Mt. Nokubi, 700 m elevation, 12 July 1987, with flowers, *S. Hatusima 42447* (KAG 161327!).

### ﻿ Hydrangea minamitanii

Taxon classificationPlantaeCornalesHydrangeaceae

(H. Ohba) Yahara stat. nov.

5B1A099D-8CB0-5B98-9D42-5868BF71F518

urn:lsid:ipni.org:names:77248599-1

Hydrangea serrata (Thunb.) Ser. var. minamitanii H. Ohba in J. Jap. Bot. 64: 199 (1989); Ohba & Akiyama, Bull. Natl. Mus. Nat. Sci., Ser. B, 39: 179 (2013). **Type.** Japan. Miyazaki Pref., Saito City, *T. Minamitani 26304* (TI).
Hortensia serrata var. minamitanii (H. Ohba) H. Ohba & S. Akiyama, J. Jap. Bot. 91: 347 (2016).

#### Japanese name.

Hyuga-ajisai.

#### Note.

*Hydrangeaminamitanii* and H.acuminatassp.acuminata often grow close, within 100 m of each other, but the former grows on wet cliffs along streams, and the latter grows on soil along forest margin. *Hydrangeaminamitanii* is distinct from *H.acuminata* in having leaves glabrous abaxially except tufted hairs at axils of lateral veins, glabrous petioles, and glabrous young shoots (Table 5). No intermediates have been discovered in localities where two species grow. *Hydrangeaminamitanii* is similar to H.acuminatasubsp.yakushimensis in growing on cliffs along streams and having leaves glabrous on both surfaces except veins and tufted hairs at axils of lateral veins, but they are distinguished by their capsule size (3.9–5.3 mm or longer in *H.minamitanii* vs. 2.2–2.7 mm in H.acuminatasubsp.yakushimensis) and flower colors (pink or white flowers vs. blue flowers). Whereas H.acuminatasubsp.yakushimensis is endemic to the Yakushima island, *H.minamitanii* is restricted to the mountains of central and eastern Kyushu, mainly in the Miyazaki Prefecture.

#### Additional specimens examined.

Japan. Miyazaki Pref.: Mt. Osuzu, 500 m elevation, 20 October 1960, with fruits, *S. Sako 3285* (KAG 161375!); ditto, 500 m elevation, 28 July 1971, with flowers, *S. Hatusima & S. Sako 32643* (KAG 161376!); ditto, 11 July 1976, with flowers, *T. Minamitani 22630* (KAG 161378!); Aya Town, 73 m elevation, 24 October 2019, with fruits, *S. Tagane 1200* (KAG 128616!).

## Supplementary Material

XML Treatment for Hydrangea acuminata

XML Treatment for Hydrangea acuminata subsp. acuminata

XML Treatment for Hydrangea acuminata subsp. yakushimensis

XML Treatment for Hydrangea acuminata subsp. australis

XML Treatment for Hydrangea minamitanii
